# Bundled care combined with animated educational videos can promote the recovery of severe pneumonia in children: A case-control study

**DOI:** 10.1097/MD.0000000000038437

**Published:** 2024-05-31

**Authors:** Wenxia Luo, Yan Liu, Caijin Wen, Xiaolong Chen, Jing Zhang

**Affiliations:** a The School of Nursing, North Sichuan Medical College; b Department of Nursing, Affiliated Hospital of Panzhihua University.

**Keywords:** animation education videos, bundled care, children, nursing effectiveness, severe pneumonia

## Abstract

In this study, we analyzed the efficacy of animated educational videos and group nursing in the treatment of severe pneumonia in children. A total of 140 patients with severe pneumonia in our hospital from October 2022 to October 2023 were selected as the research subjects, and they were divided into a control group and an observation group. The control group received routine care, while the observation group received animated educational videos and cluster nursing interventions. The treatment effects of the 2 groups of patients were compared. Clinical indicators such as body temperature recovery time, blood oxygen saturation recovery time, heart rate recovery time, consciousness recovery time, and respiratory rate recovery time were compared between the 2 groups of patients. The results showed that the temperature recovery time, oxygen saturation recovery time, heart rate recovery time and respiratory rate recovery time in observation group were significantly different from those in control group (*P* < .05). Univariate analysis showed that families with or without anxiety disorder had statistically significant differences in economic conditions, extrapulmonary complications, nursing methods and other aspects. Logistic multivariate regression analysis showed that nursing methods, extrapulmonary complications, and poor economic conditions (income < 5000) were risk factors for anxiety among family members of severe pneumonia patients, while good economic conditions (income > 5000) were protective factors. So, animated educational videos and bundled care can effectively improve the nursing effectiveness of children with severe pneumonia and promote their recovery.

## 1. Introduction

Severe pneumonia has a high incidence worldwide, affecting 156 million children each year, and has become the leading cause of death among children worldwide.^[1–3]^ In addition, severe pneumonia in children can lead to various sequelae, such as obstructive pulmonary disease, chronic bronchitis, asthma, etc, which brings a series of burdens to the family.^[4,5]^ However, in clinical care, the existing nursing methods always make the family feel that the recovery is slow and the effect is not good. Therefore, it is necessary to find new nursing methods in clinic.

Cluster nursing is a new nursing concept that combines a series of evidence-based treatment and nursing measures. A systematic review and meta-analysis by Raquel Martinez-Reviejo et al found that the implementation of bundled care reduced the number of ventilator-associated pneumonia episodes and the duration of ventilator use in adult intensive care units (ICU) patients.^[6]^ For children, a systematic review study by Teresa et al, and a study by Peter Lachman et al, found that using bundled care could prevent ventilator-associated pneumonia in neonatal ICU and reduce certain healthcare-acquired infections in pediatric and neonatal units.^[7,8]^ What more, Jing et al study showed that the implementation of Bundled care was conducive to the rehabilitation of patients with severe pneumonia.^[9]^ In previous studies, bundle nursing and animated video education have not been combined in clinical children with severe pneumonia.

This study selected children with severe pneumonia in our hospital as the research object to discuss the application effect of animated educational videos and cluster nursing in children with severe pneumonia.

## 2. Materials and methods

### 2.1. Study design

This study selected a total of 140 individuals from our hospital from October 2022 to October 2023 as the research subjects, and randomly divided them into an observation group and a control group, with 70 patients in each group. Among them, 64 were males and 76 were females.

### 2.2. Inclusion and exclusion criteria

The inclusion criteria were as follows: Meeting the diagnosis of severe pneumonia. The family members have informed consent and signed a consent form.

**Figure 1.**
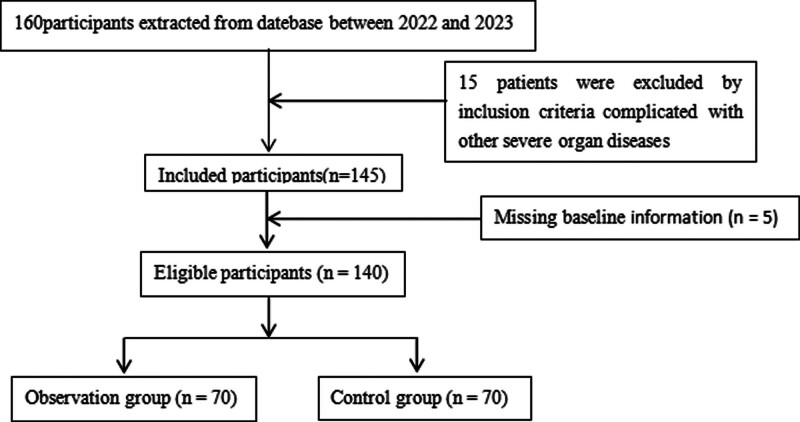
The process of selecting research subjects.

The exclusion criteria are as follows: tumor-related disease in the child; congenital heart disease. Individuals with other severe organ diseases. Premature infants. Children or family members with mental illness. The process of selecting research subjects is shown in Figure 1.

### 2.3. Nursing methods

The control group received routine nursing care, including strict aseptic procedures, observation of vital signs, maintaining appropriate humidity and temperature indoors, and measuring body temperature on this basis.

The observation group received animated educational videos and bundled care, specifically environmental care, setting up a warm ward, posting cute cartoon characters in the room, and placing some animated books and small toys. Clear communication and explanation, promptly informing family members of the patient condition and prognosis and informing them that they should participate in the treatment of the child. Paying attention to hand hygiene before and after contact with the child. Respiratory management, providing oxygen inhalation, promptly removing foreign objects from the respiratory tract, and administering nebulization inhalation and back tapping to cough up phlegm with a ventilator for children with thick sputum. The ventilator tubing was replaced every 7 days for oral care. Oral care was provided for the child in the morning and evening, including rinsing gently to avoid damage to the oral mucosa, promptly removing oral secretions, and observing for oral ulcers, stomatitis, and other conditions. Dietary care, including communicating with the hospital nutritionist to develop dietary strategies based on the preferences of the child and ensure their nutrition. When medical staff carry out treatment procedures for children, they should focus more on communication with the child, appropriately distract the child attention, encourage the child more, reduce their fear, and increase the degree of treatment cooperation. Animated educational videos were played repeatedly. Animated videos of routine nursing operations for children with pneumonia, such as injections, transfusions, and nebulization, are accompanied by animations or character images that children like. They are played in cycles during treatment breaks to enhance patient nursing coordination.

### 2.4. Outcome measures

Judging was based on efficacy. The significant therapeutic effect is that the child body temperature is normal, symptoms such as coughing and phlegm, dry or moist lung rales disappear, and the shaded areas on the chest X-ray show significant recovery; Effectively, the child body temperature is normal, and symptoms such as cough and phlegm, dry or moist lung rales are significantly improved. The X-ray chest X-ray shows some absorption of the shaded areas; Ineffectiveness refers to the absence of significant improvement or even more severe improvement in the child body temperature, clinical symptoms, or physical signs, and the absence of absorption or enlargement of the shaded areas on the chest X-ray.

The clinical indicators were as follows: body temperature recovery time, blood oxygen saturation recovery time, heart rate recovery time, consciousness recovery time, respiratory rate recovery time.

### 2.5. Statistical analysis

Statistical methods were applied to data analysis using R language software. If 2 sets of econometric data met the normal distribution and had uniform variances, 2 independent sample t-tests were used. If the variances were not uniform, a corrected t-test was used; If the normal distribution was not satisfied, the rank sum test was used. The counting data was subjected to χ^2^ test. *P <* .05 indicated a statistically significant difference.

## 3. Results

### 3.1. Basic patient data

We continuously recruited 140 children with severe pneumonia who were admitted to our hospital from October 2022 to October 2023. The basic information of these patients is shown in Table 1. There was no statistically significant difference between the control group and the observation group in terms of age, gender, relationship with the patients, economic conditions, payment method, degree of education, etc (*P *> .05, Table 1).

**Table 1:** Basic clinical data.

	Level	The control group (n = 70)	The research group (n = 70)	*P*
Age (mo) (median [IQR])		40.50 [32.25, 48.00]	44.00 [32.00, 52.75]	.448
Sex (%)	Male	34 (48.57)	30 (42.86)	.6108
	Female	36 (51.43)	40 (57.14)	
Relationship with the patients (%)	Father	18 (25.71)	22 (31.43)	.5746
	Mother	52 (74.29)	48 (68.57)	
Economic conditions (%)	Less than 3000 yuan	41 (58.57)	37 (52.86)	.1759
	3001 to 5000 yuan	18 (25.71)	27 (38.57)	
	More than 5000 yuan	11 (15.71)	6 (8.57)	
Payment method (%)	Rural medical insurance	46 (65.71)	33 (47.14)	.0858
	Urban medical insurance	22 (31.43)	34 (48.57)	
	Commercial Insurance	2 (2.86)	3 (4.29)	
Degree of education (%)	High school	38 (54.29)	43 (61.43)	.6909
	Below primary school	11 (15.71)	9 (12.86)	
	Junior middle school	21 (30.00)	18 (25.71)	

The comprehensive application of animated video education and cluster nursing in children with severe pneumonia is beneficial for the rehabilitation of children with severe pneumonia.

Compared with the control group, the temperature recovery time, blood oxygen saturation recovery time, heart rate recovery time and respiratory rate recovery time of the observation group were shortened (*P *< .05) (Table 2).

**Table 2:** Comparison of clinical symptoms between the 2 groups.

	The control group (N = 70)	The research group (N = 70)	*P*
Body temperature recovery time	10.2 (3.03)	7.08 (1.85)	<.001
Recovery time of blood oxygen saturation	8.03 (2.00)	6.43 (1.49)	<.001
Heart rate recovery time	12.3 (2.65)	8.97 (2.56)	<.001
Consciousness recovery time	12.3 (2.40)	10.3 (2.18)	<.001
Respiratory rate recovery time	7.87 (1.83)	5.86 (1.36)	<.001

Logistic regression single factor analysis and Logistic multiple regression analysis of influencing factors of family anxiety in children with severe pneumonia.

The results of univariate analysis showed that there were statistically significant differences (*P *< .05) in economic conditions, extrapulmonary complications, and nursing methods among families of severe pneumonia children with or without anxiety disorder. In addition, Logistic multivariate regression analysis showed that nursing methods, extrapulmonary complications, and poor economic conditions (income < 5000) were risk factors for anxiety among family members of severe pneumonia patients, while good economic conditions (income > 5000) were protective factors (*P *< .05) (Table 3).

**Table 3:** Logistic regression single factor analysis and Logistic multiple regression analysis of influencing factors of family anxiety in children with severe pneumonia.

Characteristic	Logistic regression univariate analysis				Logistic multivariate regression analysis					
	level	Without anxiety	Anxiety	*P*	B	SE	Wald	OR	95% CI	*P*
n		42	98							
Age1 (%)	<36 mo	13 (30.95)	35 (35.71)	.6						
	>36 mo	29 (69.05)	63 (64.29)							
Sex (%)	Male	19 (45.24)	45 (45.92)	>.9						
	Female	23 (54.76)	53 (54.08)							
Relationship with the patients (%)	Father	10 (23.81)	30 (30.61)	.4						
	Mother	32 (76.19)	68 (69.39)							
Economic conditions (%)	≤ 3000 yuan	24 (57.14)	54 (55.10)							
	3001–5000 yuan	8 (19.05)	37 (37.76)	.12	1.196	0.594	4.502	3.31	1.07, 11.3	.044
	More than 5000 yuan	10 (23.81)	7 (7.14)	.034	0.803	0.772	1.08	0.45	0.09, 1.95	.3
Payment method (%)	Rural medical insurance	24 (57.14)	55 (56.12)							
	Urban medical insurance	16 (38.10)	40 (40.82)	.8						
	Self-paying	2 (4.76)	3 (3.06)	.7						
Degree of education (%)	Below primary school	22 (52.38)	59 (60.20)							
	High school	7 (16.67)	13 (13.27)	.5						
	Junior middle school	13 (30.95)	26 (26.53)	.5						
Extrapulmonary complications (%)	No	40 (95.24)	32 (32.65)	<.001						
	Yes	2 (4.76)	66 (67.35)		4.186	0.819	26.09	65.7	16.3, 461	<.001
Nursing method (%)	Usual care	29 (69.05)	41 (41.84)	.004						
	Comprehensive care	13 (30.95)	57 (58.16)		1.674	0.53	9.903	5.33	1.96, 16.1	.002

## 4. Discussion

Pneumonia is a common respiratory infection in children, and the causes of childhood pneumonia vary, with different clinical manifestations. Sometimes, delayed diagnosis can delay treatment.^[10–12]^ The concept of cluster nursing refers to a set of nursing interventions implemented for a certain type or individual patient, each of which has been clinically proven to improve patient outcomes. The application of cluster intervention has become increasingly common in foreign countries, and cluster care is widely used in emergency and critical care.^[13]^ Some studies have found that cluster nursing can bring better satisfaction prognosis for severe patients and surgical patients.^[14–16]^ In addition, studies have found that can lead to better treatment for children.^[17]^ We recommend the use of animated video education combined with bundled care intervention in children with severe pneumonia, which is an innovative way of care.

In this study, compared with the control group, the observation group showed shorter recovery times for body temperature, blood oxygen saturation, heart rate, consciousness, and respiratory rate. Jing et al found that bundled care can accelerate the recovery of patients with severe pneumonia, which is similar to this study.^[9]^

Compared to traditional care methods, bundled care combines animated educational videos to care in a way that suits the needs and interests of the child. This not only improves the child recovery time, but also relieves the child negative emotions of nervousness and fear.^[18]^ Some scholars have found that similar studies can effectively reduce their family anxiety and strengthen the family support system. Our study found that univariate analysis showed that families with or without anxiety disorder had statistically significant differences in economic conditions, extrapulmonary complications, nursing methods and other aspects. Logistic multivariate regression analysis showed that nursing methods, extrapulmonary complications, and poor economic conditions (income < 5000) were risk factors for anxiety among family members of severe pneumonia patients, while good economic conditions (income > 5000) were protective factors. The results of this study further confirmed the application of bundled care combining animated educational videos to care in the diagnosis and treatment of severe pneumonia patients, effectively solved the problems in the treatment process, and strengthened the communication between nurses and children and their families. The anxiety of the child family was alleviated and adverse events that affected the treatment effect were avoided.

In summary, the combination of animated video education and bundled nursing interventions can effectively improve the treatment effectiveness of children with severe pneumonia, improve their condition, promote their early recovery. The combination of these nursing measures reflects humanistic care. However, there are also shortcomings in this study. The study is a single center and the sample size collected is relatively small. In future studies, the research scope can be expanded.

## Author contributions

**Conceptualization:** Wenxia Luo, Jing Zhang.

**Data curation:** Wenxia Luo, Yan Liu, Caijin Wen.

**Formal analysis:** Yan Liu, Caijin Wen.

**Investigation:** Xiaolong Chen.

**Methodology:** Xiaolong Chen.

**Software:** Caijin Wen, Xiaolong Chen.

**Supervision:** Xiaolong Chen.

**Writing – original draft:** Wenxia Luo, Jing Zhang.

**Writing – review & editing:** Wenxia Luo, Jing Zhang.
